# Effect of Chitosan Inclusion and Dietary Crude Protein Level on Nutrient Intake and Digestibility, Ruminal Fermentation, and N Excretion in Beef Heifers Offered a Grass Silage Based Diet

**DOI:** 10.3390/ani11030771

**Published:** 2021-03-10

**Authors:** Stuart F. Kirwan, Karina M. Pierce, Eleonora Serra, Michael McDonald, Gaurav Rajauria, Tommy M. Boland

**Affiliations:** School of Agriculture and Food Science, University College Dublin, W23 ENY2 Kildare, Ireland; karina.pierce@ucd.ie (K.M.P.); eleonora.serra@ucdconnect.ie (E.S.); mcdonaldm@ucd.ie (M.M.); gaurav.rajauria@ucd.ie (G.R.); tommy.boland@ucd.ie (T.M.B.)

**Keywords:** beef cattle, chitosan, crude protein, nitrogen balance, nitrogen excretion, ruminal fermentation

## Abstract

**Simple Summary:**

The level of crude protein offered to beef cattle has an impact on the level of nitrogen excreted into the environment, contributing towards ammonia emissions, which have a negative impact on terrestrial and aquatic ecosystems as well as air pollution. The aim of this study was to investigate the effects of including chitosan with different levels of crude protein on nutrient intake and digestibility, rumen fermentation, and nitrogen excretion in beef heifers offered a grass silage-based diet. Chitosan inclusion reduced nutrient digestibility, whereas feeding the high crude protein diet increased the apparent digestibility of crude protein. Feeding the high crude protein diet increased the amount of nitrogen excreted in the urine, while chitosan inclusion increased the amount of nitrogen excreted in the faeces. The chitosan used in this study had a negative effect on dry matter, organic matter, and crude protein digestibility, while feeding the high crude protein diet increased the amount of nitrogen excreted in the urine, which has a negative effect on the environment.

**Abstract:**

Nitrogen (N) use efficiency in beef cattle is low (10–20%), resulting in large amounts of N excreted into the environment. The objective of this study was to evaluate the effects of chitosan inclusion and dietary crude protein (CP) level on nutrient intake and digestibility, ruminal fermentation, and N excretion in beef heifers. Eight Belgian Blue × Holstein Friesian cross beef heifers (752 ± 52 kg BW) were used in a 4 × 4 Latin square with a 2 × 2 factorial design. Factors were dietary CP concentration—high CP, 16% (HP) or low CP, 12% (LP)—and chitosan inclusion—0 or 10 g kg^−1^ dry matter (DM) offered at 50:50 forage concentrate ratio on a dry matter (DM) basis. Apparent total tract digestibility of DM, organic matter (OM), and CP were reduced (*p* < 0.001) with chitosan inclusion, whereas offering the HP diets increased apparent total tract digestibility of CP (*p* < 0.001). Offering the HP diets increased urinary N excretion (*p* < 0.001), while chitosan inclusion increased N excretion in faeces (*p* < 0.05). Ruminal pH was increased with chitosan inclusion (*p* < 0.01). There was a CP × chitosan interaction for rumen ammonia (NH_3_) concentrations (*p* < 0.05). Including chitosan in the HP diets increased ruminal NH_3_ concentration while having no effect on the LP diets. Urinary N excretion was increased with increased levels of CP, but chitosan inclusion increased the quantity of N excreted in the faeces.

## 1. Introduction

Agriculture, especially the livestock sector, faces increasing pressure to reduce its impact on the environment (EU) 2016/2284 [1]. Nitrogen (N) excreted into the environment as a result of agricultural practices contribute towards nitrous oxide (N_2_O) emissions, a major greenhouse gas; ammonia (NH_3_), one of four transboundary gases; and the contamination of ground water through the leaching of nitrate (NO_3_^−^) [2]. During the period 2002–2016, significant increases in NH_3_ emissions were observed over several of the world’s major agricultural regions [3]. Approximately 80% of global NH_3_ emissions are a result of anthropogenic sources (animal and crop agriculture) [4]. In Ireland, agriculture is responsible for 99.1% of NH_3_ emissions, with the application of animal manures accounting for 90% of the total figure [5]. Since the abolition of EU milk quotas in 2015, NH_3_ emissions from agriculture have increased by 1.6% as a result of increased cattle numbers and urea application [5]. As part of the National Emissions Ceilings (NEC) Directive (2016/2284/EC) [6], Ireland is obliged to reduce its annual NH_3_ emissions to 104 kt by 2020, while current figures are estimated at 119 kt per annum [5].

Due to a number of different factors [7], N-use efficiency in beef cattle is low at 10–20% [8], resulting in large amounts of ingested N being excreted in urine and faeces, with urinary N accounting for 60–80% of total N excretion [9]. Urinary N is of the greatest concern, as the majority of N in urine is in the form of urea [10]. The rate of transformation of urea in urine to NH_3_ is much faster compared to N excreted in faecal matter, a process that requires the enzyme urease, which is in abundance in faecal matter [11,12]. The relationship between dietary crude protein (CP) or N intake and N excretion in the urine is far stronger (R^2^ = 0.74) than faecal N (R^2^ = 0.21) [13] and strategies that reduce CP in the diet are generally associated with a reduction in urinary N output [14,15,16].

Recently, chitosan, due to its biodegradability, antimicrobial, and nontoxic properties, has received much attention for its diverse applications in medicine and food preservation [17,18,19]. Chitosan (*N*-acetyl-D-glucosamine polymer) is a natural biopolymer formed from the deacetylation of chitin [20]. Chitin is the second most abundant organic compound on earth next to cellulose—it is found in the cell walls of lower plants and the exoskeletons of some arthropods and crustaceans [21].

The inclusion of chitosan in ruminant diets has been shown to alter ruminal fermentation, with chitosan shifting the fermentation pattern towards a more energy-efficient pathway when included in vitro [22,23,24]. Goiri et al. [25] and Dias et al. [21] noted reductions in ruminal NH_3_ concentrations with chitosan inclusion in sheep and beef cattle diets. Studies involving dairy cows and beef cattle observed that chitosan inclusion shifted the volatile fatty acid (VFA) production pattern from acetic acid to propionic acid, thereby decreasing the acetic to propionic acid ratio [26,27]. In addition to changes in ruminal fermentation, some authors noted chitosan increased apparent digestibility of dry matter (DM), CP, and neutral detergent fibre (NDF), while having no effect on nutrient intake [26,27]. However, Mingoti et al. [28] and Dias et al. [21] observed similar results in apparent digestibility of DM and CP, while simultaneously observing reductions in DM, CP, and NDF intake. To date, the effects of chitosan inclusion on ruminal fermentation has focused mainly on total mixed ration (TMR) consisting of corn silage [26,27,28,29,30], with no research investigating the effects of chitosan inclusion on ruminal fermentation in beef cattle fed grass silage.

It was hypothesised that offering chitosan to beef cattle will alter N metabolism within the rumen, increase CP digestibility in low CP diets, and in turn reduce N excretion.

The objective of this study was to evaluate the effects of chitosan inclusion with two levels of dietary CP on nutrient intake and digestibility, ruminal fermentation, and N excretion in beef heifers offered 50:50 grass silage (GS) concentrate ratio.

## 2. Materials and Methods

All procedures described in this experiment were approved by the Animal Research Ethics Committee (AREC) at University College Dublin (UCD) and conducted under experimental license from the Health Products Regulatory Authority (HPRA) (approval number: AE18982/P121) under the European directive 2010/63/EU and S.I. No. 543 of 2012. Each person who carried out procedures on experimental animals during this experiment were authorised to do so by means of individual authorisation from the HPRA. This experiment was conducted at UCD Lyons Research Farm, Celbridge, Naas, Co. Kildare, Ireland, W23 ENY2 (53°17′56″ N, 6°32′18″ W).

### 2.1. Experimental Design and Dietary Treatments

Eight Belgian Blue × Holstein Friesian cross beef heifers (*Bos taurus* strain) with an initial body weight of 752 ± 52 kg, surgically fitted with permanent ruminal cannula (100 mm i.d.) (Bar Diamond Inc., Parma, ID, USA) 575 d previously to facilitate sampling of rumen contents, were used in a replicated 4 × 4 Latin square design with a 2 × 2 factorial arrangement of treatments (*n* = 8). Factors were dietary CP level, high (16%) CP (HP) versus low (13%) CP (LP), with or without chitosan inclusion (10 g kg^−1^ DM) offered at 50:50 forage concentrate ratio on a DM basis (Table 1). Diets were offered ad libitum to ensure daily refusal of 100 g kg^−^^1^.

Chitosan was >90% degree of deacetylation, moisture 8.5%, pH 8.5, ash <1%, viscosity 40 mPa/s (A and Z Food Additives Co., Ltd., Zhejiang, China).

Diets and chitosan inclusion were offered once daily as a TMR at 08:00 using a Calan Data Ranger (American Calan, Northwood, NH, USA). The GS used during this experiment consisted of predominantly perennial ryegrass (*Lolium perenne* L.). The crop was felled during the early booth stage of vegetation (growth stage 410; [31]), wilted for 16 h, harvested with a precision chop forage harvester (mean particle length 50 mm), and ensiled under black polythene cover without the use of an additive. Prior to the commencement of the experiment, samples of silage were taken for forage analysis (Agri-Food and Biosciences Institute, AFBI-Hillsborough, Large Park, Hillsborough, Co. Down, NI, USA) using NIRS (FOSS NIR systems 5000; FOSS UK, Warrington, Cheshire, UK).

**Table 1:** Ingredient composition and chemical composition of dietary treatments.

Protein Level ^1^	HP		LP	
Chitosan Inclusion ^2^	+	−	+	−
Ingredient composition, % DM				
Grass silage	50	50	50	50
Rolled barley	26.75	26.75	31.55	31.55
Protein mix ^3^	20.50	20.50	5.7	5.7
Soyhulls	−	−	10	10
Molasses	1.5	1.5	1.5	1.5
Mineral mix ^4^	1.25	1.25	1.25	1.25
Chemical composition, % DM				
Dry matter %	35.95	35.79	35.58	35.39
Crude protein	16.38	16.30	13.27	13.30
RDP ^5^	11.10	11.10	9.25	9.25
RUP ^5^	5.28	5.28	3.80	3.80
Neutral detergent fibre	36.77	36.03	39.03	37.54
Neutral detergent fibre *forage*	51.24	51.24	51.24	51.24
Acid detergent fibre	22.20	21.89	24.52	23.91
Starch	11.96	11.68	13.77	14.10
Ether extract	2.87	2.49	2.12	2.59
Gross energy, MJ/kg	16.65	16.62	16.44	16.27

^1^ HP, high CP (16%); LP, low CP (13%). ^2^ Chitosan inclusion 10 g kg^−1^ DM. ^3^ Rapeseed meal + maize distillers grains 50:50. ^4^ Vitamins/minerals consisted of the following: calcium carbonate, sodium chloride, mono-dicalcium, phosphate, and (sugar) beet molasses. Additives per kg: Vit A (retinol) (E672) 200,000 IU, Vit D_3_ (cholecalciferol) (E671) 40,000 IU, Vit E (ali-rac-aipha-tocopheryl acetates) (3a700) 1000 IU. Compounds of trace elements: basic cobalt (II) carbonate monohydrate (3b302) 175 mg, copper sulphate pentahydrate 12,000 mg, ferric oxide (E1) 4790 mg, calcium iodate anhydrous (E2) 794 mg, manganous oxide (E5) 3226 mg, zinc oxide (E6) 5556 mg, sodium selenite (E8) 100 mg. Constituents: crude ash 96%, calcium 20%, phosphorus 2%, sodium 15%, copper added 3000 mg, selenium added 45 mg. ^5^ Calculated using NRC (2001) [32] model based on actual composition of feeds.

Each experimental period consisted of a 14 d dietary adjustment period where the animals were fed their respective diets using a Calan Broadbent controlled feeding system (American Calan, Northwood, NH, USA), followed by a 10 d experimental period where the animals were housed in metabolism stalls (1.4 × 1.8 m). During this period in the metabolism stalls, animals were allocated the first 3 d for acclimatization, followed by a 5 d N-balance study and 2 d rumen sample collection. While in the metabolism house, each animal was assigned to their own individual stall for the duration of the experiment with ad libitum access to water.

### 2.2. Data and Sample Collection

During the N-balance study, all animals were fitted with a specially constructed harness system to facilitate the separate collection of urine and faeces. This allowed the urine to flow through a plastic pipe into a plastic container, which contained 150 mL of 25% (*v*/*v*) sulphuric acid to prevent microbial degradation and the loss of volatile N as NH_3_. Total faeces were collected in trays behind each animal. Urine and faeces were weighed daily following morning feeding.

Samples of concentrates (HP and LP) were collected weekly, while GS and TMR samples were collected daily and later pooled per treatment and per animal for each experimental period. Samples were dried at 55 °C for 48 h for chemical analysis with additional samples frozen and stored at −20 °C for later total N analysis. Each morning during the N-balance study, faeces were mixed per animal and a 5% faecal sample collected. These samples were then split, a 10% sample was taken and frozen to −20 °C for subsequent chemical analysis, with the remaining sample dried at 55 °C for 240 h in a forced air oven. Dried and fresh samples of faeces were composited on an animal basis for each N-balance period. Urine samples were collected each morning with a 2.5% volume sample frozen to −20 °C.

On day 1 and 5 of each N-balance period, blood samples were collected by jugular venepuncture at 16:00 into blood collection tubes containing lithium heparin (REF: 367526, BD-Plymouth, UK), centrifuged at 1600× *g* for 20 min at 4 °C for plasma extraction. These samples were then stored at −20 °C pending analysis for plasma urea nitrogen, total protein, and creatinine concentrations.

Rumen fluid samples were collected on day 9 and 10 while in the metabolism house via the cannula for pH, NH_3_, and VFA determination. Samples were collected at 0, 1, and 2 h, and then every subsequent 2 h postfeeding for a total of 48 h. Rumen fluid was collected using a collection tube (#RT, Bar Diamond, Parma, ID, USA) and 60 mL disposable syringe. At each timepoint, 50 mL of rumen fluid from five different sites within the rumen (anterior dorsal, anterior ventral, medial ventral, posterior dorsal, and posterior ventral sac of the rumen) was collected via cannula and pooled for each animal. The pH was immediately measured (Orion 3 Star pH Benchtop Meter, Thermo Scientific, Waltham, MA, USA) and a 4 mL sample was collected using an automatic pipette and mixed with 1 mL TCA (500 g L^−1^ *w*/*v* trichloroacetic acid) prior to storage at −20 °C for subsequent VFA and NH_3_ analysis.

### 2.3. Chemical Analysis

Samples of TMR, concentrates, GS, and faeces were dried at 55 °C for 48 h in a forced air oven, ground in a hammer mill fitted with a 2 mm screen (Lab Mill, Christy Turner, Suffolk, UK), and stored for DM determination. The DM content of samples was determined after drying overnight at 105 °C (minimum 16 h) (method 930.15; AOAC [33]). Ash concentrations were determined by complete combustion in a muffle furnace (Nabertherm Gmbh, Lilienthal, Germany) at 550 °C for 5.5 h (method 942.05; AOAC [33]). Starch was determined using the Megazyme Total Starch Assay procedure (method 920.87; AOAC [33]) (product no: K-TSTA; Megazyme International Ireland LTD, Wicklow, Ireland; [34]). The N content of the feed and faeces samples were determined using the micro-Kjeldahl technique (method 920.87; AOAC [33]). Samples were weighed and placed into flasks for block digestion (unit model no. 435, Buchi, Postfach, Switzerland). Once digested, the samples were distilled (model no. 323, Buchi) into 50 mL boric acid (20 g L^−1^ *w*/*v*) containing Tashiros indicator before titration. The N content of the urine was determined using a LECO FP 528 instrument (Leco Corp., St. Joseph, MI, USA) (method 990.03; AOAC [33]). The apparent digestibility (%) of nutrients (DM, OM, CP, NDF, and starch) were calculated according to the following equation (intake and output of nutrients in kilograms):

Apparent nutrient digestibility = (1 − (faecal nutrient/total nutrient intake)) × 100 (1)

Neutral detergent fibre and acid detergent fibre (ADF) were determined by the method of Van Soest et al. [35], adopted for the use in the ANKOM^TM^ 220 Fibre Analyzer (ANKOM^TM^ Technology, Macedon, NY, USA). Concentrate samples were analysed with a thermos-stable α-amylase and 20 g of sodium sulphite (NaSO_3_) was added to neutral detergent solution (NDS), while GS and faeces samples were analysed with NDS only. Neutral detergent fibre and ADF are expressed inclusive of residual ash. Gross energy of feed and faeces samples were determined by bomb calorimetry (Parr 1281 Bomb Calorimeter, Parr Instrument Company, Moline, IL, USA). Ether extract was determined using Soxtex instruments (Tecator, Hoganas, Sweden) and light petroleum ether in feed samples only (method 920.85; AOAC [33]).

Rumen fluid samples were thawed for 16 h at 4 °C and centrifuged at 1800× *g* for 10 min at 4 °C. One ml of supernatant was drawn off and diluted 1 in 5 with distilled water (dH_2_O) and centrifuged at 1800× *g* for 15 min at 4 °C. From this, 200 µL supernatant was drawn off and NH_3_ concentrations were determined using the phenolyhpochlorite method of Weatherburn [36]. For VFA analysis, a further sample containing 250 μL of supernatant was drawn off into a separate test tube and diluted with 3.75 mL of dH_2_O and 1 mL of internal standard (0.5 g 3-metyl-*n*-valeric acid in one litre of 0.15 M oxalic acid). Following centrifuging for 5 min, 260× *g* at 21 °C, a sample was filtered through a 0.45 micrometre (μm) filter (Cronus syringe filter PTFE 13 mm; SMI-LabHut Ltd., Maisemore, Gloucester, UK) into a four ml GC vial (Thermo Scientific, Langerwehe, Germany) and frozen at −20 °C until VFA analysis. One μL of sample was injected via an auto sampler on a Varian gas chromatograph (GC) 3800 with a 25 m × 0.53 mm i.d. megabore column (coating CP-Wax 58 (FFAP) − CB (no. CP7614), (Varian, Middelburg, The Netherlands). The initial injector temperature was 75 °C, rising immediately to 95 °C, temperature increased at a rate of 3 °C/min to 200 °C (held for 50 s). Nitrogen was used as a carrier gas. The pressure of the column was held at 2.3 psi and the column rate was 8.1 mL/min.

Blood plasma samples were analysed for glucose, urea, creatinine, and total protein. Plasma urea was determined using the enzymatic kinetic method, (kit no. RX SERIES UR 3825), creatinine using colorimetric method (kit no. RX SERIES CR3814), total protein using biuret reagent (kit no. RX SERIES TP 4001), glucose using the hexokinase method (kit no. RX SERIES GL 3816). All test kits were sourced from Randox Laboratories Ltd. (Antrim, Northern Ireland, UK). All blood analyses were carried out using a clinical blood analyser (RX Imola analyser RX4900; Randox Laboratories Ltd.).

### 2.4. Statistical Analyses

Data were analysed using the PROC MIXED procedure of Statistical Analysis Software (SAS v9.4, Inst. Inc., Cary, NC, USA) for a 4 × 4 Latin square design with a 2 × 2 arrangement of treatments. Normal distribution and homogeneity of variance were analysed using the UNIVARIATE procedure. Data that were not normally distributed were transformed by raising the variable to the power of lambda. The appropriate lambda value was obtained by conducting a Box–Cox transformation analysis using the TRANSREG procedure of SAS. The model accounted for chitosan inclusion and CP level; the interaction of CP level × chitosan inclusion as a fixed effect; and square, period within square, and animal within square as random effect.

Ruminal data collected at different times after feeding were analysed using the PROC MIXED procedure for repeated measures. The model contained the same fixed effects as before, except that time after feeding and its interaction with the main effects were included.

Effects were considered significant at *p* < 0.05. When significant differences were detected, difference among treatment means and treatment by time point interaction were tested using Tukey’s multiple comparison test.

For each variable, analysed data were subjected to the following covariate structures: unstructured, variance components, compound symmetric, and toeplitz. The covariance structure that yielded the smallest Schwarz’s Bayesian criterion value was considered the most desirable for analysis.

## 3. Results

### 3.1. Nutrient Intake and Digestibility

The effect of CP level and chitosan inclusion on nutrient intake and digestibility are presented in Table 2. Crude protein level and chitosan inclusion had no effect on DMI (*p* > 0.05). Chitosan inclusion decreased the apparent total tract digestibility of DM, OM, and CP (*p* < 0.01), while having no effect on apparent total tract digestibility of NDF (*p* > 0.05). Animals offered HP diets had increased apparent total tract digestibility of CP (*p* < 0.001).

### 3.2. Nitrogen Intake and Output

Nitrogen intake ranged from 262.1 g d^−1^ to 325.8 g d^−1^ and was higher for animals offered HP diets (*p* < 0.001) versus those offered LP diets. Chitosan inclusion had no effect on N intake (*p* > 0.05). Animals that were offered HP diets excreted more N in urine compared to those offered LP diets (*p* < 0.001), with chitosan inclusion having no effect on urinary N excretion (*p* > 0.05). Furthermore, CP level had no effect on total N excreted in the faeces (*p* > 0.05), whereas including chitosan in the diet increased total N excreted in the faeces (*p* < 0.05). The animals that were offered HP diets excreted more total N (*p* < 0.001) compared to LP diets, while including chitosan in the diet had no impact on total N output (*p* > 0.05).

Feeding HP diets resulted in a higher proportion of N excreted in the urine (*p* < 0.001; 62.33 v 52.92%), while chitosan inclusion had no effect. Animals that were offered LP diets excreted a higher proportion of N in faeces compared to animals that were offered HP diets (*p* < 0.001), with no difference observed compared to the animals that were offered chitosan. Feeding HP diets increased the nitrogen recovered in the urine (*p* < 0.01), while higher proportions of N were recovered in the faeces with LP diets (*p* < 0.001) and chitosan inclusion (*p* < 0.01).

### 3.3. Blood Metabolites

Blood plasma urea was higher for animals offered HP diets (*p* < 0.01), while plasma creatinine was higher for animals offered LP diets (*p* < 0.05). No differences were observed for blood glucose levels and total protein between the two levels of CP offered (*p* > 0.05). Chitosan supplementation had no effect on any of the blood metabolites measured (*p* > 0.05).

### 3.4. Rumen Fermentation Parameters

**Figure 1:**
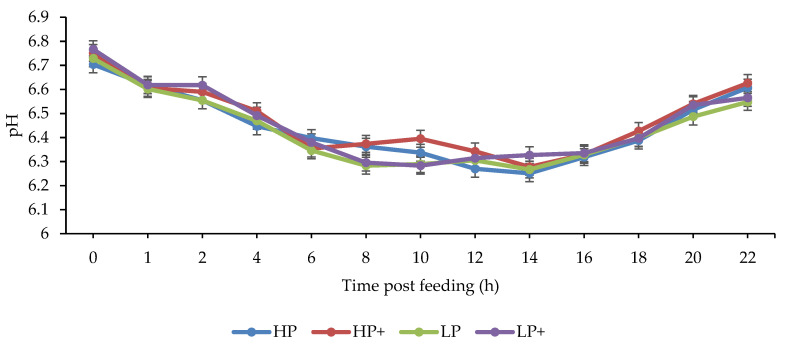
Effect of chitosan inclusion and level of crude protein on ruminal pH. Crude protein *×* chitosan *p* > 0.05; crude protein *p* > 0.05; chitosan *p* < 0.01; time after feeding *p* < 0.001.

The effect of chitosan inclusion and dietary crude protein level on rumen fermentation parameters are presented in Table 3. The level of CP that was offered had no effect on ruminal pH (*p* > 0.05), whereas animals that were offered chitosan had a higher ruminal pH (*p* < 0.01). Similar diurnal changes in pH were observed in all experimental diets (*p* < 0.001), with gradual decreases in ruminal pH observed until 14 h post feeding (Figure 1).

**Figure 2:**
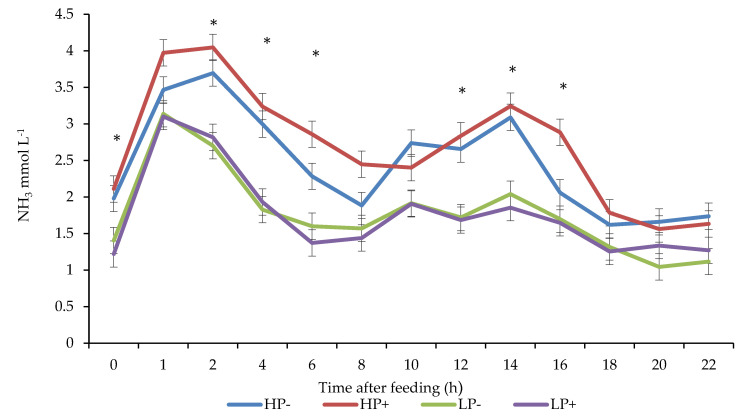
Effect of chitosan inclusion and level of crude protein on ruminal ammonia concentrations. * Denotes significance at time points between HP and LP. Crude protein × chitosan *p* < 0.01; crude protein *p* < 0.01; chitosan *p* < 0.05; time after feeding *p* < 0.001.

There was a CP × chitosan interaction for rumen NH_3_ concentrations (*p* < 0.01). Animals offered HP with chitosan included had higher ruminal NH_3_ concentrations, whereas chitosan had no impact on ruminal NH_3_ concentrations in animals that were offered LP diets. There was a CP × time interaction for rumen NH_3_ concentrations (*p* < 0.01). Ruminal NH_3_ concentrations for animals offered HP diets peaked 2 h postfeeding, while the concentrations for animals offered LP diets peaked 1 h postfeeding (Figure 2).

**Figure 3:**
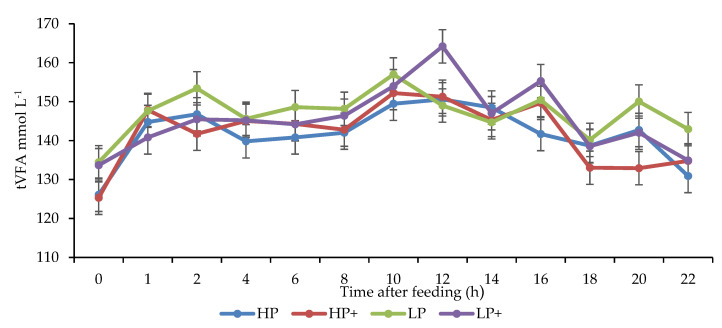
Effect of chitosan inclusion level and crude protein on ruminal total volatile fatty acid concentrations. Crude protein × chitosan *p* > 0.05; crude protein *p* < 0.001; chitosan *p* > 0.05; time after feeding *p* < 0.001.

Animals offered LP diets had higher concentrations of ruminal total VFA and acetic acid (*p* < 0.001), whereas those offered HP diets had higher concentrations of propionic acid (*p* < 0.001) (Figure 3). Crude protein level or chitosan inclusion had no effect on ruminal butyric acid concentrations (*p* > 0.05). The animals offered LP diets had a higher acetic to propionic acid (Ac:Prop) ratio compared to the animals offered HP diets (*p* < 0.001), while including chitosan in the LP diets tended to decrease the Ac:Prop ratio (*p* < 0.10). Crude protein level and chitosan inclusion had no effect on ruminal valeric acid, iso-valeric acid, and iso-butyric acid concentrations (*p* > 0.05).

## 4. Discussion

### 4.1. Nutrient Intake and Digestibility

The inclusion of chitosan in the diet reduced DM, OM, and CP digestibility, however this did not result in an alteration of DMI. This reduction in nutrient digestibility was likely due to the antimicrobial action of chitosan against ruminal microbes (protozoa and fibrolytic bacteria) [24,37]. Protozoa play an important role in protein degradation in ruminants [38], with defaunation usually resulting in decreased protein degradation [39]. Several hypotheses have been proposed as the mode of action for chitosan. The widely accepted theory is the polycationic nature of chitosan, due to the positive charges of the protonated amino groups (NH_3_^+^), allows it to interact with the negatively charged outer membrane of numerous micro-organisms, causing extensive alterations to the cell surface, leading to leakage of intracellular substances, resulting in cell death [40]. The outer peptidoglycan layer is more accessible in Gram-positive than in Gram-negative bacteria, of which most of the fibrolytic bacteria belong [17]. Goiri et al. [22,23] reported reductions in OM digestibility in vitro with chitosan inclusion, signifying activity towards cellulolytic bacteria, while Belanche et al. [20] observed a decrease in protozoal activity and rumen cellulolytic bacteria [24] responsible for the decrease in feed degradation and fermentation rate. Reducing the solubility of chitosan (<85% deacetylated) and its inclusion rate can diminish the negative impact on feed digestibility [20]. In the current study, the average daily intake of chitosan was 10 g kg^−1^ DM with a degree of deacetylation >90%. Studies where no effect or increased nutrient digestibility was observed had an average daily intake of chitosan <7.75 g kg^−1^ DM with a degree of deacetylation ≤86% [21,26,27,28,29,30].

### 4.2. Nitrogen Intake and Output

There was no effect of feeding chitosan on N intake in this study. Similar results were observed by [28,30] when included in diets offered to lactating dairy cows, whereas in a dose response study [21], chitosan quadratically affected DMI as a result of a higher intake of CP and increases in nutrient digestibility. Intake of N not used by ruminal microorganisms in microbial protein synthesis is degraded to NH_3_, metabolised to urea in the liver, and excreted in the urine. The manipulation of protein degradation in the rumen or efficient use of N in the rumen is the most cost-effective strategy to control N losses to the environment [41]. In this study, chitosan inclusion had no effect on urinary N excretion, whereas the higher the N intake associated with feeding, the higher the level of CP, which resulted in an increase in urinary excretion for those offered the HP diets. Feeding the HP diets would have supplied a higher percentage of rumen-degradable protein (RDP) compared to those offered the LP diets, resulting in excess N for microbial protein synthesis [42]. In diets where low concentrations of CP are fed, the proportion of urea produced in the liver that is returned to the rumen via blood and saliva increases, resulting in lower proportions of urea excreted via the urine [43]. Furthermore, the initial body weight of these animals indicated that these animals were mature in nature, had finished growing, and were in their finishing phase. Chitosan inclusion and the level of CP had no effect on retained N g d^−1^ (data not shown), highlighting that N intake does not interfere with muscle deposition in mature animals [44].

Faecal N is primarily of microbial origin, with lesser amounts of undegraded feed protein and endogenous secretions [45]. The increase in faecal N excretion associated with the animals that were offered chitosan is likely associated with the decrease in DM, OM, and CP digestibility. Previously, Mingoti et al. [28] found that including chitosan in the diet of lactating dairy cows reduced faecal N excretion and they concluded that this was as a result of better utilization of N, which reached the small intestine as a result of changes in rumen fermentation caused by chitosan, correlating with improvements in protein digestibility. However, Goiri et al. [25] reported a negative impact of chitosan on NDF digestibility in sheep and reduced VFA concentrations in faecal samples, suggesting that chitosan exercised the same antimicrobial action in the cecum as in the rumen, but to a greater extent.

### 4.3. Rumen Fermentation Parameters

The pH of the environment is vital to the antibacterial activity of chitosan. According to Kong et al. [17], when pH is below the molecules pK_a_ (6.3–6.5), chitosan becomes polycationic, which causes electrostatic interaction between the chitosan and the anionic components of the microorganism’s surface, while on the other hand, hydrophobic and chelating effects are responsible for antibacterial activity of chitosan when the environment is above the pK_a_. Rumen pH is a critical factor in the normal and stable functioning of the rumen because of its profound effect on microbial populations and fermentation products, and on physiological functions of the rumen. The difference observed in ruminal pH was relatively minor and would likely not be biologically significant. Cellulolytic bacteria in the rumen are responsible for fibre digestion within the rumen and as ruminal pH starts to fall below 6.2 their activity starts to diminish [46]. The negative correlation between ruminal pH and total VFA concentration highlights the pH-reducing potential of VFA accumulation in the rumen [47]. There was no difference observed in the concentration of individual VFA or total VFA concentrations from the inclusion of chitosan in the diet. However, nutrient digestibility was decreased with the inclusion of chitosan, thereby reducing the VFA production potential in the diet. Ammonia has a high pKa value (9.21) and as a consequence, virtually all NH_3_ is present in the rumen as NH_4_^+^. Production of NH_3_ in the rumen can assist in the regulation of ruminal pH through the disposal of NH_4_^+^. Therefore, the extra supply of NH_3_ from the degradation of amine groups in chitosan may explain the increase in ruminal pH associated with the inclusion of chitosan.

The higher ruminal NH_3_ concentrations associated with feeding the HP diet were expected because of the increased dietary percentages of RDP [42]. As dietary CP increases, there is greater deamination of amino acids released from protein degradation, which elevates NH_3_ [48]. The increase in iso-valeric acid associated with the LP diets are a consequence of the deamination and decarboxyl-ation of the branch-chained amino acids [49]. The addition of chitosan to the HP diets increased NH_3_ concentrations, as was similarly reported by Araújo et al. [26], where increases in NH_3_ concentrations were observed in steers fed chitosan. The increased concentrations of ruminal NH_3_ in the HP+ diet suggest that this was likely due to an extra supply of NH_3_ from the degradation of amine groups in chitosan and a lower uptake of NH_3_ by the rumen microbes, rather than increased proteolysis [24]. Kang-Meznarich and Broderick [50] reported 1.94 to 5 mmol L^−1^ to be the optimum level of ruminal NH_3_ concentration adequate for microbial synthesis and fibre digestion, suggesting the levels of ruminal NH_3_ produced in the LP diets were below optimum, which might explain why no differences were observed in ruminal NH_3_ concentrations between the two LP diets.

Previous studies found that chitosan inclusion in ruminant diets increased ruminal propionic acid concentrations [21,27], while Araújo et al. [26] observed decreases in ruminal acetic acid coupled with increases in propionic acid concentrations as a result of increased nutrient intake and digestibility. The shift in fermentation products within the rumen may be as a result of the degradation of chitosan within the rumen, with the remaining carbon skeleton used by certain bacteria [51]. Though chitosan inclusion had no effect on ruminal VFA profiles in the current study, the negative effect on nutrient digestibility may have potentially affected VFA production as a result of inefficient eating and chewing efficiency [52]. In contrast to the previous studies mentioned, the Ac:Pr was substantially higher, reflecting the high NDF contribution from the GS offered, influencing both VFA concentrations and the feeding behaviour of the animals [53]. However, the level of CP did affect the ruminal VFA profiles in this study. Feeding the higher level of CP resulted in lower ruminal acetic acid and higher propionic concentrations. Feeding the HP diets would have supplied a higher percentage of RDP [42], which has been shown to increase propionic acid and decrease acetic acid concentrations in the rumen [54].

## 5. Conclusions

Increasing protein supplementation excess to requirements can result in elevated urinary N excretion, which has negative environmental consequences. The inclusion of chitosan showed no potential in reducing N excretion, while having a negative effect on nutrient digestibility. As chitosan is not a single compound, but rather a series of different compounds that differ in degree of acetylation and other physiochemical characteristics, further studies are necessary to determine if chitosan has a role in modifying rumen fermentation.

## Figures and Tables

**Table 2:** Effect of chitosan inclusion and dietary crude protein level on dry matter intake, nutrient digestibility, nitrogen excretion, and blood metabolites in beef heifers offered grass silage-based diet.

Crude Protein ^1^	HP		LP		SEM	CP	CHI	CP×CHI
*Chitosan* ^2^	+	−	+	−				
DMI (kg d^−1^)	12.31	13.14	12.14	12.33	0.373	0.206	0.183	0.395
*Apparent total tract digestibility %*								
Dry matter	67.50	69.95	67.22	69.29	0.818	0.427	<0.001	0.745
Organic matter	69.97	72.27	70.03	71.78	0.504	0.675	<0.001	0.589
Crude protein	68.83	71.47	61.36	64.38	0.009	<0.001	0.003	0.826
NDF ^3^	48.00	50.59	49.08	50.59	1.313	0.688	0.133	0.689
*Nitrogen*								
Intake (g d ^1^)	323.8	327.9	262.3	261.9	0.009	<0.001	0.850	0.819
Output (g^−1^)								
Urine	166.1	159.8	106.1	113.8	0.009	<0.001	0.942	0.444
Faecal	100.4	93.5	101.9	93.8	0.003	0.797	0.041	0.865
Total	266.5	253.3	208.1	207.6	0.010	<0.001	0.502	0.534
*% total excreted* ^4^								
% urine	62.15	62.51	51.09	54.76	1.790	<0.001	0.272	0.366
% faecal	37.85	37.49	48.91	45.24	1.790	<0.001	0.272	0.366
*Nitrogen recovery* ^5^								
Urine	0.52	0.49	0.41	0.44	0.029	0.008	0.976	0.291
Faecal	0.31	0.28	0.39	0.36	0.009	<0.001	0.003	0.826
*Blood metabolites*								
Urea ^6^	5.48	5.31	4.08	3.88	0.147	<0.001	0.209	0.933
Creatinine ^7^	116.9	116.7	123.6	119.5	2.145	0.031	0.312	0.371
Total Protein ^8^	81.45	81.63	79.91	81.84	1.419	0.636	0.462	0.534
Glucose ^6^	3.60	3.56	3.61	3.57	0.078	0.911	0.611	0.956

^1^ HP, high CP (16%); LP, low CP (13%). ^2^ Chitosan inclusion 10 g kg^−1^ DM. ^3^ NDF, neutral detergent fibre. ^4^ % total excreted = [urine, faeces output (g/d)/Total N output (g/d) × 100. ^5^ N recovery = N out [faeces, urine (g/d)]/N intake (g/d). ^6^ mmol L^−1^. ^7^ µmol L^−1^.^8^ g L^−1^.

**Table 3:** Effect of chitosan inclusion and dietary crude protein level on rumen fermentation parameters in beef heifers offered a grass silage-based diet.

Crude Protein ^1^	HP		LP		SEM	CP	CHI	CP × CHI
Chitosan ^2^	+	−	+	−				
pH	6.47	6.44	6.45	6.43	0.025	0.163	0.002	0.870
mmol L^−1^								
NH_3_	2.69 ^a^	2.45 ^b^	1.75 ^c,d^	1.78 ^d^	0.084	<0.001	0.023	0.004
Acetic	111.2	110.7	115.3	117.3	1.62	<0.001	0.497	0.240
Propionic	14.26	14.36	13.70	13.44	0.234	<0.001	0.564	0.200
Butyric	9.24	9.33	9.00	9.25	0.206	0.338	0.355	0.633
Iso-butyric	1.87 ^a^	1.75 ^a^	1.68 ^b^	1.86 ^a,b^	0.113	0.490	0.792	0.052
Valeric	2.23	2.23	2.20	2.21	0.054	0.259	0.799	0.849
Iso-valeric	3.51	3.48	3.66	3.61	0.092	0.474	0.930	0.956
Total VFA ^3^	142.2	141.8	145.6	147.1	1.98	<0.001	0.640	0.449
Ac:Pr ^4^	8.51 ^a^	7.80 ^a^	9.07 ^b^	9.58 ^b^	0.586	<0.001	0.403	0.005

^a,b,c,d^ Different superscript letter within a row indicates significance (*p* < 0.05). ^1^ HP, high CP (16%); LP, low CP (13%). ^2^ Chitosan inclusion 10 g kg^−1^ DM. ^3^ VFA, volatile fatty acids. ^4^ Acetic: Propionic = ratio of acetic acid to propionic acid (acetic ÷ propionic).

## Data Availability

The data presented in this study are available on request from the corresponding author.
